# Acute Respiratory Distress Syndrome Associated With Clopidogrel in a Young Male Patient

**DOI:** 10.3389/fmed.2019.00038

**Published:** 2019-03-08

**Authors:** Stephanie M. Roses, Thomas Christianson, Keith Dombrowski

**Affiliations:** Department of Neurology, Duke University, Durham, NC, United States

**Keywords:** clopidgrel, plavix, ARDS (Acute respiratory distress syndrome), aneurysm, subarachanoid hemorrhage

## Abstract

**Background:** Clopidogrel is a commonly prescribed antiplatelet drug in patients with stents and histories of arterial vascular disease. It generally has a favorable side effect profile with increasing bleeding risk as the main concern as an adverse event.

**Case Presentation:** A 19-year-old previously healthy male presented to the neurological intensive care unit with a subarachnoid hemorrhage requiring a flow diverting stent to secure the aneurysm. The patient was stable for 2 weeks and had no changes to management or medication within 48 h of antiplatelet therapy. Within hours of first-time dosing of clopidogrel, the patient experienced a syncopal episode and dyspnea. He was difficult to arouse and using accessory muscles to breath with an otherwise benign exam. He was hypoxic with bibasilar crackles requiring bilevel positive airway pressure (BiPap). Imaging showed bilateral pulmonary edema and he was diagnosed with moderate acute respiratory distress syndrome (ARDS). Infectious, cardiogenic, and contrast-induced ARDS were ruled out. Upon cessation of clopidogrel, his pulmonary function and mental status improved.

**Conclusions:** This is the first reported case of a young and immunocompetent patient's severe pulmonary edema leading to acute respiratory distress syndrome in association with first- time dosing of clopidogrel.

## Background

A 19-year-old male with a remote history of asthma was brought to the a university-affiliated tertiary care medical center emergency department by emergency services after he was found obtunded at home. He had an influenza-negative upper respiratory infection, treated with oseltamivir due to flu positive contacts, for 7 days prior to admission. The day prior to admission, he called his parents and reported worst-of-life headache. He stopped responding to messages the next day, and the police department was called. He was found obtunded in his apartment. He was admitted to the intensive care unit with a Hunt-Hess 2, modified Fisher 4, subarachnoid hemorrhage with intraventricular extension secondary to rupture of a bilobed saccular aneurysm of the P2 segment of the right posterior cerebral artery. Hospital day 3 he underwent a right sub-temporal craniotomy for placement of two clips to the aneurysm, leaving a 4 mm residual aneurysm. On hospital day 4, the patient developed symptomatic vasospasm which was supported by Transcranial Doppler data. This resolved with institution of a milrinone infusion, and he remained neurologically intact the following 2 weeks in the intensive care unit. On hospital day 14 he underwent an angiogram to evaluate the residual aneurysm. This revealed a new outpouching of aneurysm adjacent to the clip, needing a flow diverting stent for management.

The patient was subsequently loaded with 150 mg of clopidogrel and started on 75 mg daily. The next morning, the patient reported feeling dizzy and short of breath while walking. Upon return to his room he was somnolent, hypotensive and using accessory muscles to breathe with an oxygen saturation of 75% with bibasilar crackles. He was quickly transitioned to BiPAP with settings as follows: fraction inspired oxygen (FiO2) 60%, inspiratory positive airway pressure 12 mmHg, expiratory positive airway pressure 10 mmHg, and his mental status improved. A norepinephrine infusion (0.01–0.03 mcg/kg/min) was required for roughly 12 h to maintain mean arterial pressure > 65 mm Hg. An arterial blood gas taken after 2 h of BiPAP revealed an arterial partial pressure of oxygen of 61 mmHg, for a P/F ratio (arterial partial pressure of oxygen/fraction inspired oxygen) of 102. On physical exam, the patient was difficult to arouse but was oriented to person, place, time, and situation and otherwise had a benign exam.

**Figure 1:**
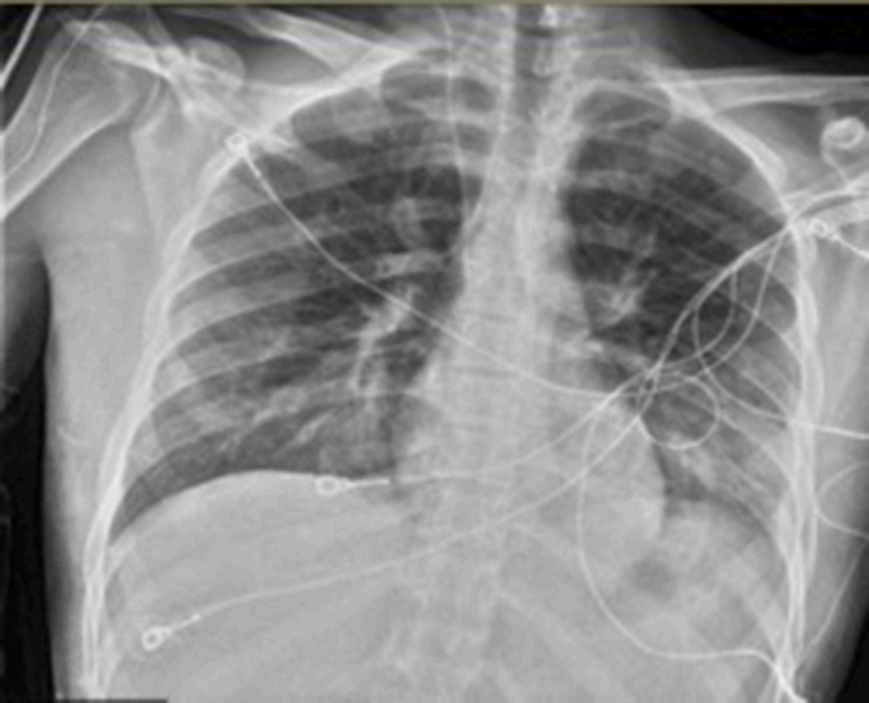
Chest radiograph immediately following syncopal episode and hypoxia.

The only laboratory abnormality was a white blood cell count of 28.4 K/μL, up from 7.5 K/μL 13 h prior. A chest X-ray showed diffuse bilateral infiltrates without cardiomegaly (see Figure 1).

**Figure 2:**
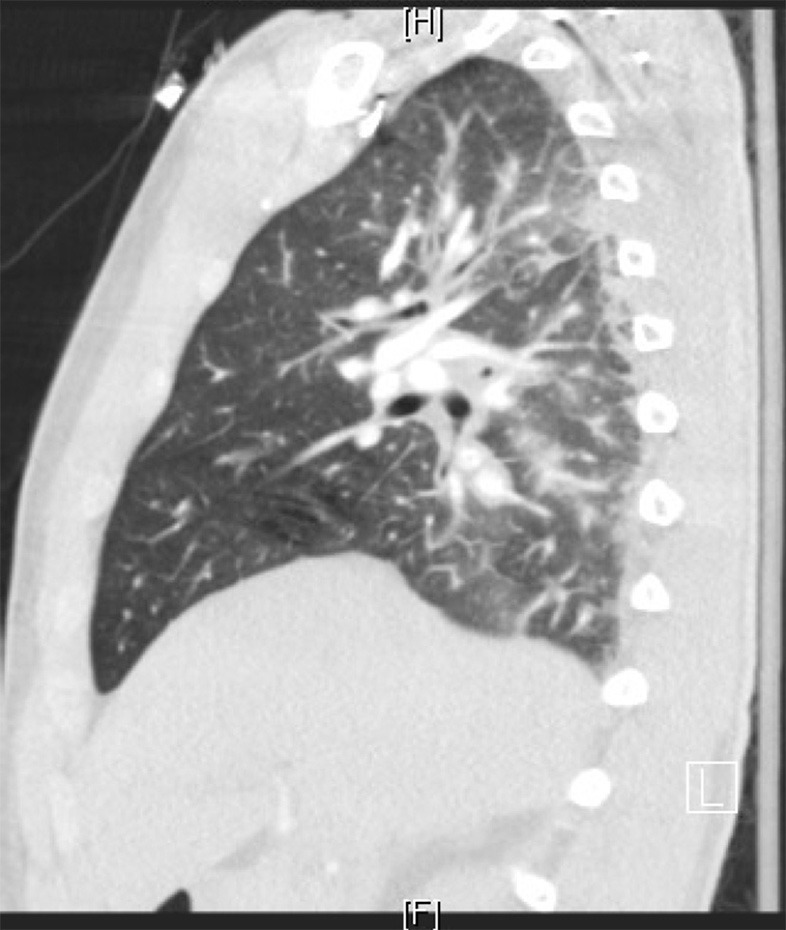
High resolution CT sagittal view of left lung and right heart border. It showed intralobular septal thickening with ground glass opacities consistent with pulmonary edema. Bilateral lower lobe bronchial wall thickening with bronchial debris and consolidative opacities were present. It did not show evidence of pulmonary embolus.

**Figure 3:**
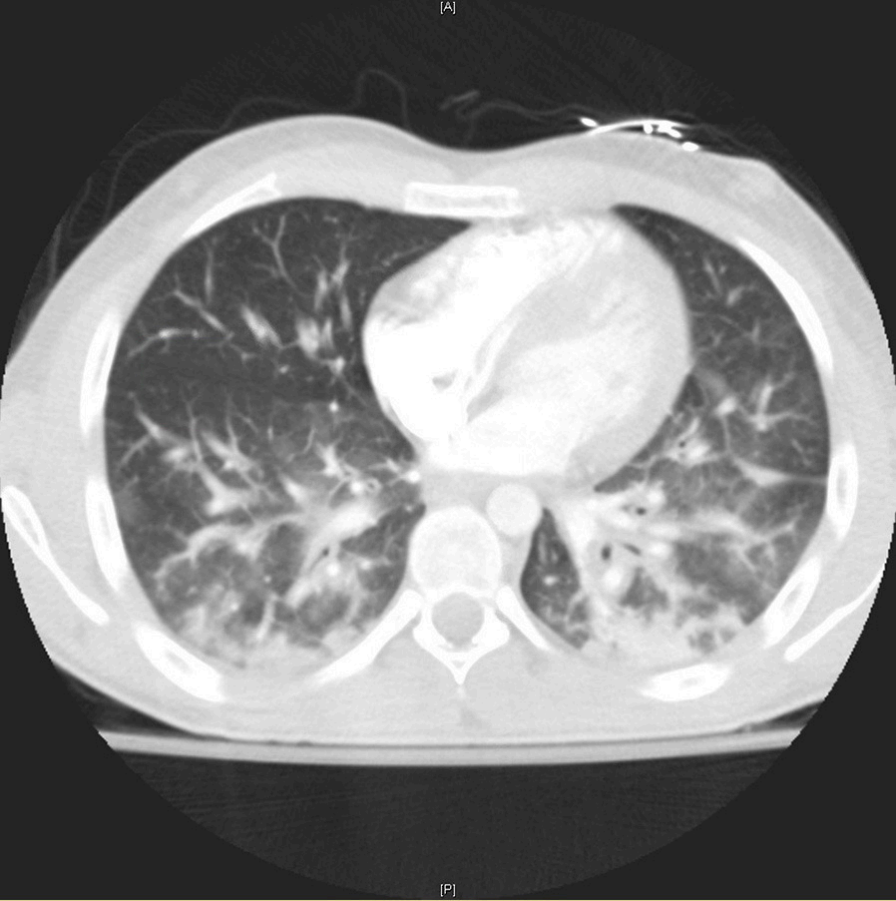
High resolution coronal view of lungs through the lung window showing bilateral intralobular septal thickening with ground glass opacities, bronchial wall thickening and consolidative opacities.

The patient was started on vancomycin and ceftriaxone for a presumed pneumonia. CTA chest revealed significant pulmonary edema without a focal consolidation or pulmonary embolism (see Figures 2, 3). A transthoracic echocardiogram was normal and unchanged from previous study 15 days prior with fully mobile and normal valves, normal chamber diameters and volumes, a calculated ejection fraction of 57%, and positive saline contrast study. Sputum, blood, and urine cultures had no growth. Clopidogrel was the only recent intervention so it was discontinued. Within 21 h the leukocytosis dropped to 12.3 K/μL and antibiotics were stopped.

## Discussion and Conclusions

Infectious Disease was originally consulted and agreed that an infectious etiology was likely. However, the next day the consulting team doubted their initial assessment as the patient's course rapidly improved in a manner not consistent with nosocomial pneumonia. His white blood cell count dropped from 28 to 12.3 K/μL 12 h after antibiotics were administered with no growth on blood or sputum culture. Afebrile the entire hospitalization, he showed no signs of infection besides radiographic chest findings and leukocytosis which occur together in many inflammatory processes, including ARDS. The leukocytosis with left shift and 88.6% neutrophils was attributed to stress demargination. There were no measured eosinophils.

The patient met Berlin criteria for moderate ARDS, though only 2 mm Hg above the severe ARDS cut-off (Table 1). The lack of hemoptysis and radiographic evidence did not support interstitial respiratory hemorrhage. His normal cardiac function and chemistries did not support fluid overload or heart failure. Allergy and Immunology were consulted in light of the possibility that this idiopathic ARDS was directly associated with the only addition to intervention within 72 h of symptom onset: Clopidogrel administration several hours before his flash pulmonary edema. We also considered contrast-induced ARDS but the patient tolerated multiple contrast loads prior to and after this event without issue. Fat embolism was unlikely since he was 2 weeks removed from the last invasive intervention and the patient had a benign skin exam throughout his hospitalization, normal hemoglobin, and normal platelet numbers. Air embolism was unlikely again because he was so far removed from surgery and no there was no evidence of focal tissue damage in the heart, lungs, or brain. While changes in mental status and alertness are not part of the Berlin Definition of ARDS, it is a common symptom of hypoxemia and not specific to any diagnosis on the differential.

**Table 1:** Acute respiratory distress syndrome (1).

Timing		Within 1 week of known clinical insult or new or worsening respiratory symptoms
Chest imaging		Bilateral opacities- not fully explained by effusions, lobar/lung collapse, or nodules on chest radiograph or CT
Origin of edema		Respiratory failure not fully explained by cardiac failure or fluid overload
		Need objective assessment (e.g., Echocardiography) to exclude hydrostatic edema if no risk factor present
**OXYGENATION**		
	Mild	200 mm Hg < Pa0_2_ / Fi0_2_ ≤ 300 mm Hg with PEEP or CPAP 5 em H_2_0
	Moderate	100 mm Hg < Pa0_2_ / Fi0_2_ ≤ 200 mm Hg with PEEP or CPAP 5 em H_2_0
	Severe	Pa0_2_ / Fi0_2_ ≤ 100 mm Hg with PEEP5 em H_2_0

Known adverse reactions to clopidogrel and its derivative, ticlodipine, include thrombotic thrombocytopenic purpura and hemorrhage. Typical allergic reactions to clopidogrel include pruritic macular, erythematous, confluent rash starting on the trunk or face, angioedema, and anaphylaxis. ARDS and pulmonary edema are not reported in the Federal Drug Administration's overview of clopidogrel and meta-analyses of associated clinical trials (2, 3). In the CAPRIE trial, fewer patients reported dyspnea in the clopidogrel with aspirin group than the placebo with aspirin group, with no difference in attrition in either group (4). In a meta-analysis, antiplatelet therapy in critically ill patients was associated with lower incidence of ARDS, particularly in those with predisposing conditions such as high-risk surgery, trauma, pneumonia, and sepsis (5). This meta-analysis looked at a broad spectrum of antiplatelet therapies and not just clopidogrel or ticlodipine.

Clopidogrel is metabolized via two main pathways in the liver. Eighty-five percentage of circulating metabolites arise from an esterase-mediated hydrolysis of clopidogrel into inactive compounds.

The CYP450 enzyme pathways metabolize the remaining clopidogrel into its active thiol metabolite over two steps. *In vivo*, the CYP450 enzyme pathway is mediated by CYP3A4, CYP2C19, CYP1A2, and CYP2B6. There are known differences in clopidogrel metabolism leading to adverse events in those with variants of the CYP2C19 enzyme, though none reported are non-cardiogenic pulmonary edema (6–8). The active thiol metabolite rapidly and irreversibly binds platelet receptors, inhibiting platelet aggregation. While unchanged clopidogrel has a serum peak at 45 min, the active thiol compound's half-life is 6 h after a single 75 mg oral dose.

There exist known variants in CYP450 enzymes that lead to differences in individual metabolism of drugs in these pathways, especially noted in CYP2C19 variants. Caucasian populations show an increased risk of adverse events and reduced platelet response in poor metabolizers. In the Ashkenazi Jewish population, such as our patient, unique combinations of ultra-rapid metabolizer and poor metabolizer alleles have been described with little clinical data (9–13). However, proponents of pharmacogenetics do suggest that in certain cases, like that of a patient with a personal or family history of unusual drug reactions, genetic testing may help determine which antiplatelet therapy is most appropriate (10, 14).

Our patient's rapid improvement more closely fit with clearance of clopidogrel, with a 6 h half-life. According to the Naranjo adverse drug reactions probability scale criteria, our patient's score of 7 means it is probable that clopidogrel caused his ARDS (see Table 2) (15). He quickly improved after discontinuation of clopidogrel and, once stable, was placed on prasugrel with no difficulties. He successfully underwent his planned drug-eluting stent placement and was discharged shortly thereafter.

**Table 2:** Naranjo Adverse Drug Reaction Probability Scale (14).

**To assess the adverse drug reaction, please answer the following questionnaire and give the pertinent score**			
	**Yes**	**No**	**Do not know**
1. Are there previous conclusive reports on this drug reaction?	**1**	0	0
2. Did the adverse event occur after the suspected drug was administered?	**2**	−1	0
3. Did the adverse reaction improve when the drug was discontinued or a specific antagonist was administered?	**1**	0	0
4. Did the adverse reaction reappear when the drug was re-administered?	2	−1	**0**
5. Are there alternative causes (other than the drug) that could have on their own caused the reaction?	−1	**2**	0
6. Did the reaction reappear when a placebo was given?	−1	1	**0**
7. Was the drug detected in the blood (or other fluids) in concentrations known to be toxic?	1	0	**0**
8. Was the reaction more severe when the dose was increased or less severe when the dose was decreased?	1	0	**0**
9. Did the patient have a similar reaction to the same or similar drugs in any previous exposure?	1	**0**	0
10. Was the adverse event confirmed by objective evidence?	**1**	0	0
Total	5	2	

*This case results in a 7 on the scale, or probable reaction due to the drug in question. The ADR is assigned to a probability category from the total score as follows: “Definite” if the overall score is 9 or higher, “probable” for a score of 5–8, “possible” for 1–4 and “doubtful” if the score is 0. Corresponding scores for this case are bolded*.

The only previous case report of non-cardiogenic pulmonary edema possibly due to clopidogrel administration was in an elderly gentleman in Turkey in 2007 (16). Within 48 h of angioplasty and clopidogrel initiation, a 71 year-old otherwise healthy patient was admitted for shortness of breath with dyspnea, tachycardia, and widely distributed pulmonary rales bilaterally. Arterial blood gas showed normal blood pH and normal partial pressure of carbon dioxide with low partial pressure of oxygen (57 mm Hg) and oxygen saturation (91%). Chest radiograph demonstrated diffuse bilateral interstitial and alveolar infiltrates with bilateral pleural effusion. High resolution CT ruled out pulmonary embolus but did show localized right-sided fluid collection and bilateral ground-glass appearance of the lungs with pleural effusions and minimal pericardial effusion. Echocardiogram, complete blood count, and throat and blood cultures were unchanged from pre-operative exam. Like the present case report, there was no evidence of the typical adverse events associated with clopidogrel like pulmonary interstitial hemorrhage. The day after discontinuation of clopidogrel, the alveolar infiltrations on chest radiograph regressed over 48 h and fully disappeared on the fifth day of withdrawal.

The previous case differs from the current case in the age and medical history of the patient, though the progression of flash pulmonary edema after clopidogrel initiation to oxygen necessity and bilateral opacities without evidence of cardiogenic or fluid overload pulmonary edema is remarkably similar. The lack of other case reports or data supporting clinically significant dyspnea or pulmonary edema suggests this is a rare but potentially fatal complication in a commonly used drug. Neither the current case nor the previous Turkish case of non-cardiogenic pulmonary edema after clopidogrel tested for CYP450 enzyme variants, especially CYP2C19. The likely eastern European descent of both patients with recent genetic evidence of Turkish descent of Ashkenazi Jews in literature (17) prompt the question of whether these reactions are more widespread than reported in certain populations, and further whether these specific populations may benefit from pharmacogenetic analysis in regard to clopidogrel administration as they often do currently for warfarin (18–23).

This case report is limited in its applications to general patient care but it does represent a clinically relevant and severe probable adverse reaction to a commonly used drug. The patient's course surprised neurological intensive care providers, and allergy, immunology, and infectious disease specialists. Future testing of this patient's CYP450 enzymes and drug metabolism has been recommended to his primary care provider in light of his hospital course. This case represents a rare but severe adverse event associated with clopidogrel administration of which providers should be aware.

## Ethics Statement

The Duke Health Institutional Review Board (IRB) does not require IRB approval or a signed informed consent for case reports of one patient. However, the patient has signed a consent to the use of their data in this case report.

## Author Contributions

SR, TC, and KD all provided direct patient care as a medical student, intensive care fellow, and attending physician respectively. All authors analyzed and interpreted data on our patient's severe acute respiratory distress syndrome. SR was a major contributor in writing the manuscript.

### Conflict of Interest Statement

The authors declare that the research was conducted in the absence of any commercial or financial relationships that could be construed as a potential conflict of interest.

## Abbreviations

BiPap: Bilevel positive airway pressure
ARDS: acute respiratory distress syndrome
CTA: contrast tomography angiography
CT: contrast tomography.
